# Social Capital and Mental Health Among Black and Minority Ethnic Groups in the UK

**DOI:** 10.1007/s10903-020-01043-0

**Published:** 2020-07-04

**Authors:** Jordan Bamford, Gonnie Klabbers, Emma Curran, Michael Rosato, Gerard Leavey

**Affiliations:** 1 Queen’s University Belfast, Belfast, Northern Ireland UK; 2 Department of Health Ethics and Society, Faculty of Health, Medicine and Life Sciences, Maastricht University, Maastrichtt, The Netherlands; 3 Bamford Centre for Mental Health & Wellbeing, Ulster University, Coleraine, UK

**Keywords:** Ethnic minorities, Mental health, Wellbeing, Social capital

## Abstract

Black and minority ethnic communities are at higher risk of mental health problems. We explore differences in mental health and the influence of social capital among ethnic minority groups in Great Britain. Cross-sectional linear and logistic regression analysis of data from Wave 6 (2014–2016) of the *Understanding Society* databases. In unadjusted models testing the likelihood of reporting psychological distress (i) comparing against a white (British) reference population Indian, Pakistani, Bangladeshi and *mixed* ethnic minority groups recorded excess levels of distress; and (ii) increasing levels of social capital recorded a strong protective effect (OR = 0.94: 95% CI 0.935, 0.946). In a subsequent series of gender-specific incremental logistic models-after adjustment for sociodemographic and socioeconomic factors Pakistani (males and females) and Indian females recorded higher likelihoods of psychological distress, and the further inclusion of social capital in these models did not materially alter these results. More research on the definition, measurement and distribution of social capital as applies to ethnic minority groups in Great Britain, and how it influences mental wellbeing is needed.

## Introduction

### Background

Black and minority ethnic (BME) communities appear to be at a greater risk of psychosis compared to the white UK-born population [1–3] and rates of depressive symptoms are higher among BME groups in Europe [4]. In the UK, Pakistani men are twice as likely to report a Common Mental Disorder (CMD) when compared against white males [5, 6]. Rates of mental illness differ among BME groups and are not reflective of rates in their country of birth [7]. Explanations of raised vulnerability for mental disorders among BME populations include issues with migration, settlement and experience of racism and discrimination, poverty and adverse environmental conditions [7–9].

### Theoretical Framework

Social capital refers to those potentially positive aspects of social life and is constructed through shared networks, norms, and trust. It enables a more effective pursuit of shared objectives [10] and is commonly described as having two components: cognitive social capital—subjective factors acting to keep networks together (and measured by indicators such as trust, social support and neighbourhood satisfaction); and structural social capital—attachment to organisations such as churches and measured by attendance and strength of commitment [11]. Unlike structural social capital, cognitive social capital has been indicated as an important predictor of mental wellbeing [12]. High levels of social capital may enhance a sense of belonging and thus increase collective wellbeing [13]. Conversely, where social capital is low individuals may feel insecure and alienated.

While there is no real consensus on the relationship between social capital and mental wellbeing [14] some evidence suggests that smaller social networks, fewer close relationships, and lower perceived adequacy of social support are associated with depressive symptoms [15, 16]. BME populations experience such issues in the United Kingdom (UK) [17, 18]. While racism may have a detrimental effect on BME social capital and wellbeing [19, 20] there has been scant research that considers how social capital impacts wellbeing among BME groups in the UK [1].

## Methods

### Participants

We used a cross-sectional analysis of data drawn from Wave 6 (2014–2016) of the *Understanding Society* database, which contains representative samples of BME and white populations in the UK [21]. Understanding Society is a longitudinal survey of households in the UK [22].

### Data Collection

A detailed description of Understanding Society, sample design and the ethnic minority and migrant population sample structure has been published previously [23, 24]. Comprehensive descriptions of the techniques and methodology used is published elsewhere [25], as are sampling methodologies [26]. Data collection was conducted face-to-face via computer aided personal interviews, with additional self-completion instruments such as the General Health Questionnaire-12 (GHQ12) administered separately.

We extracted data from Wave 6 only. The final dataset used in analysis comprised 25,921 observations—a total arrived at as follows: a boosted sample (n = 4656) of ethnic minority participants in Wave 6 was excluded because they were not asked some detailed questions we relied on in this analysis; the *natural* attrition from Wave 1 to Wave 6 had been 35.4%, reducing the initial sample from 40,634 observations; and a small number of observations containing either missing values for the variables used in the analysis or where information had been gathered via proxies (less than 1%) were also excluded from analysis. Because of the relatively small amount of missing data, and large sample size it was thought unnecessary to impute this information.

## Measures

### BME Groups

Ten *ethnicity* groups were identified: white (British); white (Irish); white (other); mixed ethnicity; Indian; Pakistani; Bangladeshi; Caribbean, African, with a residual *other* category comprising minorities deemed too small to justify separate categories for analysis. The *mixed* group represents a growing group of UK citizens whose parents are each from different ethnic groups, primarily partnerships between white British and people from ethnic minority groups [27]. The white (other) group comprise those minorities who identify as both white and not British or Irish.

### Mental Health

The GHQ12 is a self-administered screening test used among respondents in community and non-psychiatric clinical settings to assess psychological distress. It has reliability coefficients ranging from 0.78 to 0.95 and good sensitivity and specificity among BME groups [28–31]. From the GHQ12 caseness (psychological distress = yes) was derived as a binary field with a cut-off point of three or more (from range 0–12) signalling distress [29, 32].

### Social Capital

The Individual components of social capital—each with responses ranging from one (strong disagreement) to five (strong agreement)—have been found to be valid elsewhere [33–35]. We summed these to give an overall score: participants were asked about their neighbourhood, and how strongly they felt about the following: the close-knit nature of their neighbourhood; the willingness of people to help neighbours; whether people in their neighbourhood can be trusted; whether people in the neighbourhood get along with each other; whether individuals belong to the neighbourhood; if they can borrow things from neighbours; and finally, if they feel similar to others in their neighbourhood. Allowing for reverse-coding the summary scale ranged from eight to forty (with higher scores indicating greater social capital). This social capital score demonstrated high internal consistency (Cronbach’s alpha = 0.84).

### Migrant and Acculturation Factors

From country of birth we derived *born in UK* (Yes/No). Acculturation and sense of assimilation was assessed via a continuous variable, *British Identity* measuring how individuals perceived the importance of being British, with responses on a scale from zero (not important at all) to ten (extremely important).

### Sociodemographic and Socioeconomic Factors

These include: age (continuous); gender; marital status (grouped as single, married/cohabiting and, as a single group, those widowed, separated or divorced); family structure; and locale of residence—summarised as urban or rural (and generated by the core Understanding Society data management team using information provided by the Office for National Statistics Rural and Urban Classification of Output Areas). Family structure comprised four categories—single (no children), in a couple (no children), in a couple (with a child), or single (with a child). Proxy indicators of socioeconomic circumstance included home ownership (yes, no), economic activity (employed, not employed, retired) and educational level. Education was classified as: primary (no GCSEs); secondary (GCSEs, A-levels or equivalent non-vocational attainment) or tertiary (degree level).

### Analysis

Analysis utilized SPSS Version 25 software (SPSS Inc., Chicago, IL, USA). Descriptive statistics for continuous variables included means, standard deviation and range, with percentages presented for categorical variables. All findings are presented for males and females separately. Independent sample T-tests for continuous variables, and Pearson’s chi-square for categorical variables determined gender differences in the population. We calculated mean differences in social capital across ethnic groups with a one-way ANOVA, and used linear regression to explore the relationship between ethnicity and social capital. Binary logistic regression examined determinants of psychological distress for the total sample and for men and women separately. Fully adjusted odds ratios (ORs) and 95% Confidence Intervals were derived. For all analyses, p-values of less than 0.05 were considered significant.

### Ethics

This study was completed in keeping with the relevant ethical and legal obligations of data usage from the UK Longitudinal Household Study: as this information is publicly available ethical approval was not sought.

## Results

More than 20% of the sample were psychologically distressed (Table 1). Eighty percent of the sample were white British, and the largest single ethnic minority group was Indian (3.1%). The mean age of the sample was 49.2 years (standard deviation (SD) 17.4) and 56% (14,432) were female. The predominant education status was High School level, 63% were employed and 74% reported owning their house. Over 65% were married or cohabiting, and 48% lived in households with more than one adult and no children. Over three quarters lived in urban areas, 88% were born in the UK and 54% professed a religious affiliation. The mean for social capital was 29.1 (SD 5.0) and for British identity—where medians were more appropriate measures—the median was 8 (range 0–11). Prevalence of psychological distress ranged from 21% in the white British population to 34% in the Pakistani population (Table 2). Gender specific differences were evident across most factors, with the exception of locale of residence, nativity and Britishness. Females were more likely than men to be younger, better educated, a single parent, report a religious affiliation, be born outside the UK and to report psychological distress; and less likely to be employed or be home owners. Additionally, women reported higher social capital levels.

**Table 1:** Descriptive statistics of study population

	All 100% (25,921)	Male 44.3% (11,489)	Female 55.7% (14,432)	p value
	% (n)	% (n)	% (n)	
*Psychological distress*				
Yes	21.6 (5608)	18.0 (2067)	24.5 (3541)	< 0.05
No	78.4 (20,313)	82.0 (9422)	75.5 (10,891)	
*Ethnicity*				
White (British)	82.0 (21,249)	82.6 (9495)	81.4 (11,754)	< 0.05
White (Irish)	1.6 (412)	1.6 (184)	1.6 (228)	
White (other)	2.4 (621)	2.2 (252)	2.6 (369)	
Mixed	1.7 (443)	1.5 (178)	1.8 (265)	
Indian	3.1 (804)	3.5 (398)	2.8 (406)	
Pakistani	2.4 (635)	2.4 (273)	2.5 (362)	
Bangladeshi	1.3 (346)	1.3 (153)	1.3 (193)	
Caribbean	1.6 (422)	1.3 (152)	1.9 (270)	
African	1.6 (420)	1.5 (167)	1.8 (253)	
Other	2.2 (569)	2.1 (237)	2.3 (332)	
*Education level*				
Primary	24.0 (6214)	24.9 (2866)	23.2 (3348)	< 0.05
Secondary	38.4 (9949)	39.4 (4527)	37.6 (5422)	
Tertiary	37.6 (9758)	35.7 (4096)	39.2 (5662)	
*Economic activity*				
Employed	63.0 (16,342)	67.1 (7714)	59.8 (8628)	< 0.05
Not employed	13.0 (3360)	8.5 (974)	16.5 (2386)	
Retired	24.0 (6219)	24.4 (2801)	23.7 (3418)	
*Owner occupier*				
Yes	73.5 (19,045)	75.3 (8648)	72.0 (10,397)	< 0.05
No	26.5 (6876)	24.7 (2841)	28.0 (4035)	
*Marital status*				
Married/cohabiting	66.3 (17,196)	70.4 (8083)	63.1 (9113)	< 0.05
Not married	19.5 (5049)	20.3 (2334)	18.8 (2715)	
Wid/sep/divorced	14.2 (3679)	9.3 (1072)	18.0 (2604)	
*Family structur*e				
Single, no children	15.3 (3978)	15.2 (1747)	15.5 (2231)	< 0.05
Single, & children	3.6( 923)	0.7 (79)	5.8 (844)	
Couple, no children	47.9 (12,410)	51.2 (5879)	45.3 (6531)	
Couple, & children	33.2 (8610)	32.9 (3784)	33.4 (4826)	
*Locale of residence*				
Urban	75.7 (19,628)	75.7 (8700)	75.7 (10,928)	0.99
Rural	24.3 (6293)	24.3 (2789)	24.3 (3504)	
*Belongs to a religion*				
Yes	53.9 (13,964)	48.3 (5544)	58.3 (8420)	< 0.05
No	46.1 (11,957)	51.7 (5945)	41.7 (6012)	
*Born in UK*				
Yes	87.8 (22,763)	88.1 (10,122)	87.6 (12,641)	0.21
No	12.2 (3158)	11.9 (1367)	12.4 (1791)	

Continuous variable	Mean (SD)	Mean (SD)	Mean (SD)	
*Age*				
Range 17–102	49.2 (17.4)	49.7 (17.6)	48.8 (17.3)	< 0.05
*Social capital*				
Range 8–40	29.1 (5.0)	28.9 (4.8)	29.3 (5.2)	< 0.05

British identity	Median (range)	Median (range)	Median (range)	
Range 0–11	8 (0–11)	8 (0–11)	8 (0–11)	

Social capital varied significantly across ethnic groups (Table 3**).** Generally males recorded stronger effect sizes than women over the range of minority groups. Compared to the white British group, white (other), *mixed*, Caribbean, African and *other* ethnic groups reported lower social capital, while white (Irish) and Pakistani groups reported higher social capital. In the stratified analyses males recorded stronger effect sizes of white (Other), *mixed*, Caribbean, African and *other* ethnicities were more likely to report lower social capital whereas white (Irish), Pakistani and Bangladeshi groups reported higher social capital levels than the white British group. Similarly, for women, low social capital was reported by mixed, Caribbean, African and *other* ethnic groups (with no groups reporting higher social capital).

**Table 2:** Levels of psychological distress, by ethnic group and sex

	All		Male		Female	
	% (95% CI)	n	% (95% CI)	n	% (95% CI)	n
White (British)	20.7 (20.1, 21.2)	4369	17.1 (16.3, 17.8)	1620	23.6 (22.9, 24.4)	2776
White (Irish)	22.3 (18.4, 26.7)	92	19.6 (14.1, 26.0)	36	24.6 (19.1, 30.7)	56
White (other)	21.7 (18.6, 25.2)	135	20.6 (15.8. 26.2)	52	22.5 (18.3, 27.1)	83
Mixed	29.3 (25.1, 33.8)	130	24.7 (18.6, 31.7)	44	32.5 (26.9, 38.5)	86
Indian	25.5 (22.5, 28.7)	205	21.6 (17.7, 26.0)	86	29.3 (24.9, 34.0)	119
Pakistani	34.0 (30.3, 37.8)	216	32.2 (26.7, 38.1)	88	35.4 (30.4, 40.5)	128
Bangladeshi	25.7 (21.2, 30.7)	89	21.6 (15.3, 28.9)	33	29.0 (22.7, 36.0)	56
Caribbean	24.6 (20.6, 29.0)	104	26.3 (19.5, 34.1)	40	23.7 (18.8, 29.2)	64
African	24.5 (20.5, 28.9)	103	16.2 (10.9, 22.6)	27	30.0 (24.5, 36.1)	76
Other	24.3 (20.8, 28.0)	138	17.3 (12.7, 22.7)	41	29.2 (24.4, 34.4)	97

Data represents proportion in group reporting distress, 95% confidence intervals and number in group

**Table 3:** Levels of social capital, by ethnic group and sex

	All (mean)	All beta (95% CI)	Males beta (95% CI)	Females beta (95% CU)
White (British)	29.18	Ref	Ref	Ref
White (Irish)	30.12	0.94 (0.45, 1.42)*	1.43 (0.73, 2.13)*	0.54 (−0.13, 1.21)
White (other)	28.54	−0.64 (−1.04, −0.24)*	−1.20 (−1.80, −0.60)*	−0.29 (−0.82, 0.24)
Mixed	27.62	−1.56 (−2.03, −1.09)*	−1.09 (−1.80, −0.37)*	−1.92 (−2.55, −1.30)*
Indian	29.02	−0.17 (−0.52, 0.19)	−0.05 (−0.53, 0.44)	−0.23 (−0.74, 0.27)
Pakistani	29.61	0.43 (0.03, 0.82)*	1.03 (0.45, 1.61)*	−0.04 (−0.58, 0.49)
Bangladeshi	29.52	0.34 (−0.20, 0.87)	0.84 (0.07, 1.61)*	−0.07 (−0.80, 0.66)
Caribbean	27.62	−1.56 (−2.04, −1.08)*	−0.96 (−1.73, −0.19)*	−1.97 (−2.59, −1.35)*
African	27.31	−1.88 (−2.36, −1.40)*	−1.14 (−1.87, −0.40)*	−2.41 (−3.05, −1.77)*
Other	28.05	−1.14 (−1.56, −0.72)*	−0.83 (−1.45, −0.21)*	−1.39 (−1.95, −0.83)*

Data represents mean scores and beta coefficients (95% CI) for each group

*p < 0.05

**Table 4:** Psychological distress, for all in sample

	Unadjusted OR (95% CI)
*Ethnicity*	
White (British)	1.00
White (Irish)	1.10 (0.87, 1.39)
White (other)	1.07 (0.88, 1.29)
Mixed	1.59 (1.29, 1.96)*
Indian	1.31 (1.12, 1.54)*
Pakistani	1.98 (1.67, 2.34)*
Bangladeshi	1.33 (1.04, 1.69)*
Caribbean	1.25 (1.00, 1.57)
African	1.25 (1.00,1.56)
Other	1.23 (1.01, 1.49)*
*Gender*	
Male	1.00
Female	1.48 (1.40, 1.58)*
*Age (continuous)*	
Range between 17 and 102	0.99 (0.989, 0.992)*
*Education level*	
Tertiary	1.00
Secondary	1.10 (1.03, 1.18)*
Primary	1.10 (1.02, 1.19)*
*Economic activity*	
Employed	1.00
Not employed	2.81 (2.59, 3.04)*
Retired	0.87 (0.80, 0.93)*
*Owner occupier*	
Yes	1.00
No	1.72 (1.61, 1.83)*
*Marital status*	
Married/cohabiting	1.00
Never married	1.54 (1.43, 1.66)*
Widowed/separated/div	1.44 (1.33, 1.57)*
*Family structure*	
One adult, no children	1.00
One adult, with children	1.84 (1.59, 2.13)*
Multiple adults, no children	0.92 (0.86, 0.99)*
Multiple adults, with children	1.15 (1.06, 1.26)*
*Locale of residence*	
Rural	1.00
Urban	1.25 (1.17, 1.35)*
*Belongs to a religion*	
Yes	1.00
No	1.04 (0.98, 1.10)
*Born in UK*	
Yes	1.00
No	1.13 (1.03, 1.23)*
*British identity*^**£**^	
Range between 0 and 10	0.96(0.95, 0.97)*
*Social capital*^**£**^	
Range between 8 and 40	0.94 (0.935, 0.946)*

Data represents unadjusted odds ratios (and 95% CI)

*P < 0.05

^£^Both *British identity* and *social capital* are represented as continuous with units ranging from low to high in the respective measures—the ORs reflect the change per unit increase: in both cases showing a protective effect with higher levels

**Table 5:** Likelihood of reporting psychological distress, stratified by gender—in a series of four incrementally adjusted models

	m1: minimally adjusted ($) OR (95% CI)	m2: m1 + socioeconomic factors (&) OR (95% CI)	m3: m2 + sociodemographic factors (£) OR (95% CI)	m4: m3 + social capital OR (95% CI)
*All persons*				
White (British)	1.00	1.00	1.00	1.00
White (Irish)	1.08 (0.85, 1.36)	1.04 (0.82, 1.32)	0.94 (0.73, 1.20)	0.99 (0.77, 1.28)
White (other)	1.01 (0.83, 1.22)	0.98 (0.80, 1.19)	1.05 (0.84, 1.30)	1.04 (0.84, 1.30)
Mixed	1.43 (1.16, 1.77)*	1.30 (1.05, 1.61)*	1.26 (1.01, 1.56)*	1.23 (0.99, 1.54)
Indian	1.27 (1.08, 1.50)*	1.26 (1.07, 1.49)*	1.39 (1.15, 1.67)*	1.40 (1.16, 1.69)*
Pakistani	1.79 (1.51, 2.12)*	1.48 (1.24, 1.76)*	1.65 (1.36, 2.00)*	1.73 (1.43, 2.10)*
Bangladeshi	1.17 (0.91, 1.49)	0.95 (0.73, 1.22)	1.06 (0.81, 1.38)	1.14 (0.87, 1.48)
Caribbean	1.20 (0.96, 1.50)	1.06 (0.85, 1.34)	1.00 (0.79, 1.27)	0.97 (0.76, 1.23)
African	1.13 (0.90, 1.42)	0.97 (0.77, 1.22)	1.04 (0.81, 1.35)	1.00 (0.77, 1.29)
Other	1.14 (0.94, 1.39)	1.05 (0.86, 1.28)	1.13 (0.91, 1.41)	1.10 (0.88, 1.38)
*Males*				
+-White (British)	1.00	1.00	1.00	1.00
White (Irish)	1.16 (0.80, 1.68)	1.09 (0.75, 1.59)	0.87 (0.89, 1.29)	0.97 (0.65, 1.44)
White (other)	1.22 (0.89, 1.66)	1.19 (0.86, 1.63)	1.07 (0.76, 1.52)	1.04 (0.73, 1.48)
Mixed	1.48 (1.05, 2.09)*	1.31 (0.92, 1.88)	1.23 (0.85, 1.78)	1.24 (0.86, 1.80)
Indian	1.28 (1.01, 1.64)*	1.33 (1.03, 1.71)*	1.29 (0.96, 1.72)	1.31 (0.98, 1.75)
Pakistani	2.15 (1.66, 2.79)*	1.97 (1.50, 2.58)*	1.93 (1.42, 2.61)*	2.11 (1.56, 2.86)*
Bangladeshi	1.22 (0.82, 1.80)	1.06 (0.70, 1.58)	1.02 (0.66, 1.57)	1.13 (0.74, 1.74)
Caribbean	1.72 (1.19, 2.47)*	1.40 (0.96, 2.04)	1.28 (0.86, 1.89)	1.28 (0.87, 1.90)
African	0.88 (0.58, 1.33)	0.75 (0.49, 1.16)	0.70 (0.44, 1.11)	0.70 (0.44, 1.11)
Other	0.96 (0.68, 1.35)	0.87 (0.61, 1.24)	0.82 (0.56, 1.20)	0.82 (0.56, 1.19)
*Females*				
White (British)	1.00	1.00	1.00	1.00
White (Irish)	1.02 (0.75, 1.39)	1.00 (0.73, 1.36)	0.97 (0.70, 1.33)	1.00 (0.72, 1.38)
White (other)	0.90 (0.70, 1.15)	0.88 (0.68, 1.13)	1.05 (0.79, 1.40)	1.06 (0.80, 1.12)
Mixed	1.40 (1.08, 1.82)*	1.28 (0.98, 1.68)	1.26 (0.96, 1.66)	1.22 (0.93, 1.61)
Indian	1.26 (1.01, 1.57)*	1.23 (0.98, 1.53)	1.45 (1.13, 1.85)*	1.46 (1.14, 1.86)*
Pakistani	1.57 (1.26, 1.96)*	1.27 (1.01, 1.59)*	1.48 (1.15, 1.90)*	1.52 (1.18, 1.94)*
Bangladeshi	1.13 (0.83, 1.55)	0.90 (0.65, 1.25)	1.06 (0.76, 1.49)	1.12 (0.80, 1.58)
Caribbean	0.99 (0.75, 1.32)	0.91 (0.68, 1.21)	0.89 (0.66, 1.20)	0.85 (0.62, 1.15)
African	1.27 (0.97, 1.67)	1.09 (0.83, 1.44)	1.28 (0.94, 1.75)	1.21 (0.88, 1.66)
Other	1.25 (0.98, 1.59)	1.15 (0.90, 1.47)	1.36 (1.04, 1.79)*	1.32 (1.00, 1.74)

Results represent odds ratios and 95% confidence Intervals

^$^For all persons—minimally adjusted for age/sex; for males and females separately, each adjusted for age

^&^Socioeconomic factors—education level; whether employment; and whether retired or not

^£^Sociodemographic factors—marital status; family structure; locale of residence; belonging to a religion; whether born in the UK; and extent of feeling a British identity

*p < 0.05

Table 4 shows results from a series of unadjusted models examining likelihood of recording psychological distress for each of the factors included in the analyses. Compared to the white British five ethnic minority groups—mixed, Indian, Pakistani, Bangladeshi, and *other*—recorded excess likelihoods, highest (OR = 1.98: 95% CI 1.67, 2.34) amongst Pakistanis. Females were more likely than males to report distress (OR = 1.48: 1.40, 1.58); as were those not employed when compared to their employed peers (OR = 2.81: 2.59, 3.04); those living in urban areas when compared to their rural peers (OR = 1.25: 1.17, 1.35); and those not born in the UK (OR = 1.13: 1.03, 1.23). Those with higher education levels were somewhat protected, as was being currently married (compared to those never married or currently not married—OR = 1.54: 1.43, 1.66 and 1.44: 1.33, 1.57 respectively). Finally, the factors tested as continuous variables all show protective effects in their respective models: psychological distress declines by 1% with increasing age (OR = 0.99: 0.989, 0.992); and 6% (OR = 0.94: 0.935, 0.946) and 4% (OR = 0.96: 0.95, 0.97) for increasing levels of social capital and increasing strength of feelings of Britishness respectively.

Table 5 shows the likelihood of experiencing psychological distress by ethnic group (compared against white British) in a series of incrementally adjusted models, ending with full adjustment for all selected characteristics. Only the results for ethnic group are presented (the full model table is available on request). In the minimally adjusted model (M1) those from Indian, Pakistani and mixed ethnicities (and, for males only, Caribbean) showed excess likelihoods for reporting distress when compared to the white (British) group. With further adjustment for sociodemographic and socioeconomic characteristics (M3) the excesses initially recorded for males, with the exception of those of Pakistani ethnicity (OR = 1.93: 95% CI 1.42, 2.61), disappeared suggesting a strong effect of sociodemographic and socioeconomic factors on levels of distress. Similarly, both Indian and Pakistani women recorded likelihoods significantly different from white British women (OR = 1.45: 1.13, 1.85 and OR = 1.48: 1.15, 1.90 respectively). In the final model (M4, also including social capital) Indian and Pakistani women maintained this effect noted above (OR = 1.46: 1.14, 1.86 and OR = 1.52: 1.18, 1.94 respectively), while for men the effect remained only for Pakistanis (OR = 2.11: 1.56, 2.86), suggesting that, in this study, social capital exerts a relatively weak independent effect in models which include sociodemographic and socioeconomic characteristics.

## Discussion

To our knowledge this is the first study to examine the influence of social capital on the mental health of a wide range of ethnic groups in the UK. Our findings suggest that, compared to their white British peers, psychological distress may be more prevalent in some (but not all) BME communities. This corroborates other studies [1, 5, 36]. In the British Psychiatric Morbidity Survey [37] common mental disorders were found in around one adult in six and were more prevalent in specific population groups. These included Black women, adults under the age of sixty who lived alone, women resident in large households, unemployed adults, those in receipt of benefits and those who smoked cigarettes. In the EMPIRIC study [5] ethnic differences in CMD prevalence were modest. After adjusting for differences in socio-economic status CMD risk was higher amongst Irish and Pakistani men aged 35–54 years, compared to white UK-born people. Higher rates of CMD were also observed among Indian and Pakistani women aged 55–74 years, compared to white women of similar age. Higher rates of psychological distress among Indian and Pakistani groups may be partly related to racism and/or disadvantage [38–41], but it is unclear why some BME communities should be more affected than others.

In this study, while unemployment is a more specific determinant of psychological distress among males, for women (additional to unemployment) more personal, culturally significant factors exert particular pressures: for example, low educational attainment, marriage (but without children), and being born outside the UK. Previous research suggests specific socio-cultural factors—influence of extended family, single women not chosen for marriage, infertility, gender of offspring and social isolation—that may be relevant to psychological distress in Pakistani women [43]. In this study, for men and women, home ownership, greater sense of British identity and higher levels of social capital were protective for mental health, suggesting that economic security and settlement in the UK (both possibly indicated by home-ownership) influence wellbeing within such communities.

These findings underscore the different experiences and concerns of women and men in minority ethnic communities, with consequences that are differentially distributed across minority ethnic groups and which could be determined by the length of settlement and the resilience of their respective communities in coping with socioeconomic adversity [44, 45]. Thus, for example, educational attainment may have wider implications for women than men in traditional communities—while women may be more restricted in relation to their wider social and educational access this, however, may also signify higher levels of integration and increased social inclusion within the group [46, 47]. Similarly, while infertility is a source of distress for many women it may carry greater resonance in more traditional communities [48].

In this study, for men and women separately and for ethnic groups, levels of social capital appear significantly associated with mental ill-health, corroborating current evidence of associations between social capital and CMDs [49]. The protective effect of social capital on mental wellbeing is in agreement with other evidence [50–53]. However, in the fully adjusted models (which included sociodemographic and socioeconomic characteristics) inclusion of the measure for social capital did not materially mitigate recorded levels of psychological distress. This underlines the importance of contextual social and political factors and how these may impact on the mental health of BME populations. This study indicates which ethnic minority groups in the UK experience a greater risk of psychological distress, indicating where investment of mental health resources are needed. Gender differences for distress among BME groups imply that appropriate interventions should be specific for men and women. The influence of social capital on mental wellbeing warrants further study. Investment into civic society type organisations, in order to build up trust and cohesion could possibly improve mental wellbeing.

## Conclusions

This study suggests that in the UK certain BME groups—especially Indian, Pakistani and Bangladeshi groups—are at an increased risk of psychological distress. Levels of social capital are high in Pakistani and Bangladeshi men, but low for Caribbean and African women. While determinants for psychological distress may differ considerably among men and women, and social capital appears to be an important determinant of mental health for both men and women and for specific ethnic groups, its effect is diluted when examined against socioeconomic and sociodemographic considerations. These findings indicate possible need for investment in community-specific public health interventions to improve sense of security and belonging particularly among minority ethnic groups.

## Strengths and Limitations

Understanding Society contains a representative sample of minority ethnic populations in the UK. The inclusive and broad conceptualisation of ethnicity and self-reporting eliminates researcher bias. Using a large representative sample aids reliability. While the GHQ12 is not a diagnostic tool, it is well validated for use with ethnic minority groups. The analysis is cross-sectional, and as such no causality can be implied. Another limitation relates to the (unvalidated) measure of social capital used—however, items used in its construction relate to a number of the constructs of social capital. Further validation is warranted.
